# Cognitive impairment and hypertension in older adults living in extreme poverty: a cross-sectional study in Peru

**DOI:** 10.1186/s12877-017-0628-8

**Published:** 2017-10-26

**Authors:** Maria Lazo-Porras, Victor Ortiz-Soriano, Miguel Moscoso-Porras, Fernando M. Runzer-Colmenares, German Málaga, J. Jaime Miranda

**Affiliations:** 1 CRONICAS Center of Excellence in Chronic Diseases, Universidad Peruana Cayetano Heredia, Av. Armendariz 497, Miraflores, 241 69 78 Lima, Peru; 2 Unidad de Conocimiento y Evidencia (CONEVID), Universidad Peruana Cayetano Heredia, Av. Armendariz 497, Miraflores, 241 69 78 Lima, Peru; 3 Aging Research Center, School of Medicine, Universidad de San Martin de Porres, Lima, Peru; 4 School of Medicine, Universidad Peruana Cayetano Heredia, Lima, Peru; 5 Medicine Service, Hospital Cayetano Heredia, Lima, Peru

**Keywords:** Cognitive impairment, Hypertension, Poverty, Peru

## Abstract

**Background:**

Previous studies have shown that hypertension is a risk factor for cognitive impairment, but whether this association is also present in extremely poor populations in Low Middle Income Countries settings remains to be studied. Understanding other drivers of cognitive impairment in this unique population also merits attention.

**Methods:**

We performed a secondary analysis using data from the “Encuesta de Salud y Bienestar del Adulto Mayor”, a regional survey conducted in an extremely poor population of people older than 65 years old from 12 Peruvian cities in 2012. The outcome variable was cognitive impairment, determined by a score of ≤7 in the modified Mini-Mental State Examination. The exposure was self-reported hypertension status. Variables such as age, gender, controlled hypertension, education level, occupation, depression and area of living (rural/urban) were included in the adjusted analysis. We used Poisson regression with robust variance to calculate prevalence ratios (PR) and 95% confidence interval (95% CI) adjusting for confounders.

**Results:**

Data from 3842 participants was analyzed, 51.8% were older than 70 years, and 45.6% were females. The prevalence of cognitive impairment was 1.7% (95% CI 1.3%–2.1%). There was no significant difference on the prevalence of cognitive impairment between the group of individuals with hypertension in comparison with those without hypertension (PR = 0.64, 95% CI 0.33–1.23).

**Conclusions:**

The association described between hypertension and cognitive impairment was not found in a sample of extremely poor Peruvian older adults.

**Electronic supplementary material:**

The online version of this article (10.1186/s12877-017-0628-8) contains supplementary material, which is available to authorized users.

## Background

People with cognitive impairment progressively lose cognitive functions such as memory, attention, language, and task execution without reaching levels of dementia or Alzheimer’s [1]. In a survey conducted in rural and urban areas of eight low middle-income countries (LMICs) the prevalence of cognitive impairment ranged from 0.8 to 4.3% [2]. The most important risk factors for developing cognitive impairment can be non-modifiable, such as genetic factors, age, female sex, educational level, socioeconomic status, lack of intellectual activity [2] and modifiable factors like depression, diabetes, smoking, and hypertension [3].

The association between cognitive impairment and hypertension becomes important, as both are conditions that affect, directly or indirectly, the independence and quality of life of older adults [4]. Hypertension has a general prevalence of 22.6% in Peru, reaching up to 26.2% in adults of 60–69 years old, and up to 36.3% in those ≥70 years old [5]. Findings from longitudinal studies show that the presence of hypertension, especially poorly controlled hypertension (systolic blood pressure ≥ 140 mmHg and diastolic blood pressure ≥ 90 mmHg), increases in 2-fold the risk for development and rapid progression of cognitive impairment in 25 years of follow-up [6–10]. The pathophysiologic mechanism of this association is not well understood, although it is known that presence of hypertension is associated with subcortical lesions, white matter lesions, lacunar infarcts and cerebral microhemorrhages, all possible causes of cognitive impairment and its progression [11].

The evidence of an association between hypertension and cognitive impairment in Peru and other LMICs is limited, especially in populations with a low socioeconomic status. Determining such associations would be important because could guide the screening and treatment of these conditions. A Mexican study has shown that people with a low socioeconomic status have higher incidence of cognitive impairment throughout their lives [12].

Previous studies in Peru have described a great gap between diagnosis, treatment, and control of hypertension [13] and reports worldwide show that the gap increases in settings with fewer resources (33% more awareness of hypertension in people with higher socioeconomic status in comparison to lower socioeconomic status) [14]. Poverty leads to less access to healthcare, less awareness, and treatment of hypertension, if an association with cognitive impairment is found, is possible that the magnitude of this association would be higher in comparison to other values found in previous studies in not deprived populations. Exploring cognitive impairment and hypertension in a population living in extreme poverty will give us supplementary information about the burden of both diseases. Furthermore, it will provide us with an insight into the extent of the problem and the association of both conditions in Peru, which could be extrapolated to other LMICs with similar conditions. Given the rapid growth of the older population, which is expected to reach 2 billion by the year 2050, [15] the the aim of this study was to determine the association between cognitive impairment and hypertension in an extremely poor elderly population in Peru. Additionally, we know that other social determinants are important in the development and progression of cognitive impairment, and this warrants their evaluation as predictors of cognitive impairment.

## Methods

### Study design and participants

We performed a secondary analysis of a public dataset from the “Encuesta de Salud y Bienestar del Adulto Mayor 2012” (Health and Welfare Survey for the Elderly 2012 – ESBAM 2012), which collected demographic, social, and economic data from older adults [16]. Data collected from ESBAM 2012 was used as the baseline of some indicators for the Peruvian social program “Pension 65”. This program began on 2012 as a government initiative to provide protection for elderly population and to provide them with a financial subsidy of 125 PEN per month per person [17].

To secure sample’s geographical representativeness, ESBAM 2012 included urban and rural participants from 111 of 184 provinces with a total population of 4242 older adults. Inclusion criteria were: households with older adults, living in extreme poverty [18] and meeting eligibility criteria for the “Pension 65” program (not having a retirement pension, severance, or widowhood by private or public entities, not having Social Health Insurance, and not being included in other social programs).

### Sampling procedures

Primary sampling units (PSU) were selected independently by province and area of residence, rural or urban. For the rural area, PSU were the populated centers, and only those with more than three households were eligible for the program. For the urban area, PSUs were predefined clusters within each stratum, i.e. states or departments. On a second stage, a random sampling was performed within each PSU to recruit participants for the survey. Data collection was performed between October and November 2012 before the commencement of participation in the government’s “Pension 65” program.

### Evaluation and measurements

#### Cognitive impairment

We defined cognitive impairment through a modified version of Mini-Mental State examination (MMSE). This instrument was modified and validated in Chile for the “Encuesta de Salud Bienestar y Envejecimiento” (Health, Welfare, and Aging survey), by the Pan American Health Organization in 1999 [19]. The MMSE contains six items with a total of 19 points and a cut-off at 13 points for the moderately educated population of Chile. A score of 13 or less was considered as cognitive impairment, with a sensibility of 93.8% and a specificity of 93.9% [19, 20]. In Peru, the ESBAM 2012 used five out of the six items of this tool. The missing item, a question introducing 5 numbers to the participant [1, 3, 5, 7, 9] and requesting the subject to repeat them in reverse order, was eliminated by the ESBAM 2012 team because of its complexity anticipating difficulties in rural areas and illiterate populations that do not know the numbers and the sequence of them. Hence, the scale with 5 items used had a maximum total of 14 points. This modified 5-item version was used previously in Mexico, in the “First Follow-up to the Evaluation of the Impact of the Program of care for Adults over 70 years old from rural areas” (“Programa 70 y más” in Spanish), but not report of the validation was found [21]. This simplified 5-item tool had a cut-off of 7 points or less to define cognitive impairment.

Also, the score of the MMSE was used as count data to performed additional analysis.

### Hypertension

Hypertension was ascertained from participant’s self-report during face-to-face interviews in which the participants were asked if they had hypertension and if it was diagnosed by a health professional. For individuals with a score ≤ 7 in the modified MMSE, a caregiver reported the information on their behalf. Furthermore, we consider an additional definition of hypertension, self-reported hypertension or among those who reported not having hypertension but had a single measure of blood pressure (BP) ≥140/90 mmHg were also considered as having hypertension in additional analysis.

### Other variables

Additional covariates were considered, such as sex, age group (65–69, 70–80 years old), educational level (illiterate, complete/incomplete primary education, higher/technical education), self-reported depression (yes/no) and employment status (employed/unemployed). In terms of additional clinical variables, among those who reported having hypertension, participant’s hypertension treatment status (treated vs untreated) was recorded and between the participants with treated hypertension, control was considered if their BP was <140/90 mmHg (controlled vs uncontrolled).

### Statistical analysis

We used chi-square tests for the bivariable analysis between cognitive impairment and hypertension, also bivariable analysis between cognitive impairment and other sociodemographic characteristics. For the multivariable analysis, we used a Poisson family distribution with log link function and robust standard error estimation to calculate crude and adjusted prevalence ratios (PR) and their respective 95% confidence intervals (95%CI), the outcome of interest was cognitive impairment and the exposure was hypertension using self-report hypertension in comparison with participants without hypertension. Also, an additional model was performed using as exposure hypertension according to blood pressure measure and in comparison with the participants with normal blood pressure measure. Variables used to adjust the models were selected using previous studies to included potential confounders available in the database [22]. For all estimations, we took into account the complex nature of the sampling and used complex survey commands accordingly. In the survey design, strata defined in the sampling were departments and clusters (PSU) were villages or populated centers, thus percentages and other calculations are weighted to correct for stratification and clustering. All analyses were performed in Stata 12 for Windows (StataCorp, College Station, TX).

### Ethics

The Ministry of Development and Social Inclusion was in charge of the development of the questionnaires and assessments [14]. The Peruvian National Institute of Statistic and Informatics was in charge of the data collection. The present study is a secondary analysis of the publicly available database and uses de-identified data. The Institutional Review Board of the Universidad Peruana Cayetano Heredia approved the protocol for this analysis.

## Results

### General characteristics and prevalence of cognitive impairment

From the 4242 participants of the ESBAM 2012, we analyzed a total of 3842 older adults for this study. 9% of the sample was not included in the statistical analyses due to missing information. We found a cognitive impairment prevalence of 1.7% (95% CI 1.3%–2.1%) and a self-reported hypertension prevalence of 24.3% (95%CI 22.8% – 25.8%). Among those who reported hypertension, 59.1% received treatment, and only 41% of them had BP controlled. Other characteristics of the study sample are shown in Table 1.

**Table 1:** Main characteristics of the sample studied

	Number	Percent^a^
Age (years)		
65–70	1849	48.2
71–80	1993	51.8
Gender		
Female	1753	45.6
Male	2089	54.4
Educational level		
None	3044	79.2
Primary or superior	797	20.8
Depression self report		
No	3131	85
Yes	553	15
Occupation		
No	1232	32.1
Yes	2610	67.9
Area		
Urban	1515	39.4
Rural	2327	60.6
Health insurance		
No	1366	35.8
Yes	2470	64.2
Cognitive impairment		
No	3721	98.3
Yes	64	1.7
Hypertension		
No	2909	75.7
Yes	933	24.3
Blood pressure ≥ 140/90 mmHg		
No	2520	65.5
Yes	1322	34.5

^a^ weighted percentages

### Association of cognitive impairment and hypertension and other characteristics of the population

We did not find an association between hypertension and cognitive impairment; the prevalence of cognitive impairment in patients without hypertension was 1.8% and 1.3% in those with hypertension (*p* = 0.335). Instead, we found an association between cognitive impairment and age (0.7% in the group between 65 and 70 years and 2.6% in the group between 71 and 80 years, *p* < 0.001), occupation, gender, educational level and living in rural area. (Table 2).

**Table 2:** Bivariate associations to cognitive deterioration

Characteristics	Cognitive impairmentn (%)^a^		p^b^
	No	Yes	
Age (years)			<0.001
65–70	1826 (99.3)	12 (0.7)	
71–80	1895 (97.4)	52 (2.6)	
Gender			<0.001
Female	1682 (97.6)	43 (2.4)	
Male	2039 (99.0)	21 (1.0)	
Educational level			0.020
None	2941 (98.1)	58 (1.9)	
Primary or superior	779 (99.2)	6 (0.8)	
Self report depression			0.664
No	3043 (98.5)	48 (1.5)	
Yes	529 (98.7)	7 (1.3)	
Occupation			<0.001
No	1144 (96.5)	42 (0.5)	
Yes	2577 (99.2)	22 (0.8)	
Blood pressure ≥ 140/90 mmHg			0.934
No	2435 (98.3)	42 (1.7)	
Yes	1286 (98.3)	22 (1.7)	
Area			0.018
Urban	1472 (97.9)	49 (2.1)	
Rural	2249 (99.0)	15 (1.0)	
Health insurance			0.363
No	1330 (98.6)	19 (1.4)	
Yes	2385 (98.2)	45 (1.8)	
Hypertension			0.335
No	2819 (98.2)	52 (1.8)	
Yes	902 (98.7)	12 (1.3)	
Blood pressure ≥ 140/90 mmHg			0.934
No	2435 (98.3)	42 (1.7)	
Yes	1286 (98.3)	22 (1.7)	

^a^ weighted percentages

^b^ Corrected for the complex sample design

### Risk factors for cognitive impairment: Hypertension and other characteristics

In the crude analysis, people with hypertension show similar prevalence of cognitive impairment when compared with those without hypertension (*p* = 0.337). After performing the regression analyses to adjust for confounders we did not find an association between hypertension and cognitive impairment. Analysis using cognitive impairment as a count variables are show in Additional file 1: Table S1.

Even using self-reported hypertension or BP measured ≥140/90 mmHg as exposure, no association was found. Also when we consider treated vs untreated and controlled vs uncontrolled no association was found (Additional file 1: Table S2).

Regarding other associated factors, there was evidence of an association in both the crude and adjusted models for age, occupation, and living in a rural area. However, for educational level and gender, the association with cognitive impairment was found in the crude model, but not in the adjusted model. (Table 3).

**Table 3:** Factors associated to cognitive impairment

	Bivariate analysis			Multiple regression^a^		
	PR	95% CI	*p*	PR	95% CI	*p*
Age (years)			<0.001			<0.001
65–70	Ref.			Ref.		
71–80	4.04	2.08–7.85		3.55	1.82–6.94	
Gender			0.001			0.097
Female	Ref.			Ref.		
Male	0.43	0.26–0.71		0.59	0.32–1.10	
Educational level			0.026			0.569
None	Ref.			Ref.		
Primary or superior	0.4	0.18–0.90		0.78	0.34–1.82	
Occupation			<0.001			<0.001
No	Ref.			Ref.		
Yes	0.24	0.14–0.44		0.27	0.14–0.54	
Area			0.020			0.003
Urban	Ref.			Ref.		
Rural	2.07	1.12–3.83		2.65	1.40–5.00	
Hypertension			0.337			0.177
No	Ref.			Ref.		
Yes	0.72	0.37–1.40		0.64	0.33–1.23	

^a^Adjusted for all the variables

## Discussion

### Main findings

Our findings show there is no association between hypertension and cognitive impairment in older adults with a low socioeconomic status from Peru. Earlier studies that looked into this topic showed controversial results with a weak association between both conditions; [23] however, recent studies have shown a more consistent correlation [6–9]. The finding in our study may reflect survivor bias since the sample is a very depressed population and is possible that people with the exposure and outcome of interest are under-represented and could be underestimating the association.

Also, a recent review of the American Heart Association stated that there is consistent information that high blood pressure in midlife is associated with an altered cognitive function in the elderly. However, the association of high BP in late life and oldest old age with cognitive function is less clear. Studying this association in an extremely poor adult population should also be informative. This statement supports the needed of more consistent evidence in old population, actual information reflects differences in the cognitive domains evaluated, the difference in study design and follow-up period [24].

Among the potential predictors evaluated we found that in people of rural residency and age between 71 and 80 years cognitive impairment was more prevalent in comparison with their counterparts in order of 40% to 594% more. Whereas participants that had an occupation had less prevalence of cognitive impairment between 46% and 76% in comparison to participants without occupation.

### Comparison to other findings

We found an association between living in rural areas and cognitive impairment; very similar findings were described in a study conducted in Spain, which determined 1.13 greater odds of cognitive impairment in people from rural areas in comparison to urban areas [25]. There are a number of possible reasons for this association, people living in rural areas have less access to good education, and the possibility of performing intellectual activities, which have consistently been associated with a lower risk for cognitive impairment [26]. Other studies have described that living in urban areas is a risk factor for cognitive impairment although they relate this to the presence of industrial solvent exposure [27].

Moreover, we found an association between unemployment and cognitive impairment. There is evidence supporting the link between active lifestyles and lower risk of cognitive impairment and dementia [28, 29]. We did not explore the relationship between the complexity of the occupation and dementia, as this is known to be a protective factor [30]. A recent study created a middle age risk score for early prediction of dementia using prospective information and found that 4 different variables related to work were associated with higher risk of dementia: working status (not working), nature of work (very monotonous), work environment (outdoors/outdoors and indoors) and physicality of work (heavy manual working). We did not include these characteristics in our analysis, but most of the population in our study performed a heavy manual working in agriculture or cattle breeding [31].

In the bivariate analysis, we found a lower prevalence of cognitive impairment in male in comparison to female; however, after adjusting for confounders, the relation between these two factors was non-significant, in contrast to other studies [25, 32, 33]. Hebert et al. obtained a similar result to ours and concluded that the higher prevalence of cognitive impairment in women was due to their longer life expectancy, which overestimated this value when compared to elderly males [34].

### Limitations

The tool used to measure cognitive impairment is a modified version of the MMSE test, which removes the attention/concentration item. A study in Japan showed that variance of the working memory factor, which includes attention/concentration, is 3.19%, and its load factor is 0.53, which corresponds to the lower variance and lower load factor in the factorial analysis [35]. It is assumed, therefore, that the measure of cognitive impairment will not be affected by the absence of the attention/concentration item in the MMSE.

The exposure used in the study was self-reported hypertension, and because in participants with cognitive impairment the information was collected from a caregiver, we could have underestimated the presence of the disease. For this reason, we did an additional analysis, using as exposure the BP measured ≥140/90 mmHg that did not found an association between cognitive impairment and hypertension.

Other potential confounders such as physical activity, obesity, smoking, and alcoholism were not available in the dataset. Not analyzing these confounders could produce a positive bias in our estimations, because these variables increase the prevalence of the exposure and the outcome.

### Application of results and next steps

Although our results show no association between cognitive impairment and hypertension, this is one of the first studies that evaluate the magnitude of both conditions in older adults with a low socioeconomic status. In a low-resource setting, this is relevant as many factors related to hypertension are different from urban areas and it can get worse in poverty conditions. We have also found other important factors associated with cognitive impairment that will help us target the screening of cognitive impairment in older adults in our setting. Finally, the world’s population over 60 years old is growing at a faster rate than the total population all over the world, especially in the less developed regions. [15]

## Conclusion

There is no association between cognitive impairment and hypertension in older adults with a low socioeconomic status. However, those who are unemployed, and living in rural areas had a higher prevalence of cognitive impairment. The burden of the cognitive impairment in this underserved population would have great consequences at individual, social and economic levels. For these reasons, early actions during life and social support can prevent some of the most important consequences of cognitive impairment.

## Additional file

Additional file 1:
**Table S1.** Analysis using cognitive impairment as a count variable. **Table S2.** Treated vs untreated and controlled vs uncontrolled. (DOCX 21 kb)

## Abbreviations

BP: Blood pressure
CI: Confindence intervals
ESBAM: Health and Welfare Survey for the Elderly by its spanish acronym
LMICs: Low middle-income countries
MMSE: Mini-mental short examination
PR: Prevalence ratios
PSU: Primary sampling units

## References

[1] Petersen RC (2011). Clinical practice. Mild cognitive impairment. N Engl J Med.

[2] Sosa AL, Albanese E, Stephan BCM, Dewey M, Acosta D, Ferri CP (2012). Prevalence, distribution, and impact of mild cognitive impairment in Latin America, China, and India: a 10/66 population-based study. PLoS Med.

[3] Etgen T, Sander D, Bickel H, Förstl H (2011). Mild cognitive impairment and dementia: the importance of modifiable risk factors. Dtsch Arzteblatt Int.

[4] Wlodarczyk JH, Brodaty H, Hawthorne G (2004). The relationship between quality of life, mini-mental state examination, and the instrumental activities of daily living in patients with Alzheimer’s disease. Arch Gerontol Geriatr.

[5] Instituto Nacional de Estadística e Informática (INEI). Prevalencia de la hipertensión arterial en personas de 50 y más años de edad. Informe Técnico N° 1 Salud Familiar. 2010 [cited 2014 Jul 30]. Available from: http://bvsper.paho.org/videosdigitales/matedu/20110131_prevalencia_hipertension_INEI.pdf?ua=1.

[6] Kilander L, Nyman H, Boberg M, Hansson L, Lithell H (1998). Hypertension is related to cognitive impairment: a 20-year follow-up of 999 men. Hypertens Dallas Tex 1979.

[7] Elias PK, Elias MF, Robbins MA, Budge MM (2004). Blood pressure-related cognitive decline: does age make a difference?. Hypertens Dallas Tex 1979.

[8] Skoog I, Lernfelt B, Landahl S, Palmertz B, Andreasson LA, Nilsson L (1996). 15-year longitudinal study of blood pressure and dementia. Lancet Lond Engl.

[9] Goldstein FC, Levey AI, Steenland NK (2013). High blood pressure and cognitive decline in mild cognitive impairment. J Am Geriatr Soc.

[10] Launer LJ, Masaki K, Petrovitch H, Foley D, Havlik RJ (1995). The association between midlife blood pressure levels and late-life cognitive function. The Honolulu-Asia aging study. JAMA.

[11] Sierra C, Doménech M, Camafort M, Coca A (2012). Hypertension and mild cognitive impairment. Curr Hypertens Rep.

[12] Zeki Al Hazzouri A, Haan MN, Kalbfleisch JD, Galea S, Lisabeth LD, Aiello AE (2011). Life-course socioeconomic position and incidence of dementia and cognitive impairment without dementia in older Mexican Americans: results from the Sacramento area Latino study on aging. Am J Epidemiol.

[13] Lerner AG, Bernabe-Ortiz A, Gilman RH, Smeeth L, Miranda JJ (2013). The “rule of halves” does not apply in Peru: awareness, treatment, and control of hypertension and diabetes in rural, urban, and rural-to-urban migrants. Crit Pathw Cardiol.

[14] Kanungo S, Mahapatra T, Bhowmik K, Saha J, Mahapatra S, Pal D (2017). Patterns and predictors of undiagnosed and uncontrolled hypertension: observations from a poor-resource setting. J Hum Hypertens.

[15] World Population Ageing. [cited 2017 Jan 28]. Available from: http://www.un.org/esa/population/publications/worldageing19502050/

[16] Results from the National Survey of Health and Wellfare of the Elderly Population. 2012 [cited 2014 Jul 29]. Available from: http://www.midis.gob.pe/dgsye/evaluacion/documentos/Resultados_de_la_ESBAM.pdf

[17] Pension 65 [Internet]. [cited 2014 Jul 29]. Available from: http://www.pension65.gob.pe/

[18] SISFOH. [cited 2014 Jul 30]. Available from: http://www.sisfoh.gob.pe/nosotros.shtml?x=1478

[19] Minimental State Examinations (MMSE) del Estudio de dementia en Chile. [cited 2014 Jul 30]. Available from: file:///F:/Bibliograf%C3%ADa/mmet%20modificado.pdf

[20] Evaluation of the mental and emotional capacity, in elderly adults. [cited 2014 Jul 29]. Available from: http://www.sld.cu/galerias/pdf/sitios/gericuba/modulo4.pdf

[21] Cuestionario individual corto. Primer seguimiento a la evaluación de Impacto del Programa de atención a adultos mayores de 70 años y más en zonas rurales. [cited 2014 Jul 30]. Available from: file:///C:/Documents%20and%20Settings/Casa/Mis%20documentos/Descargas/55211_cuest_individual_AM_2008.pdf

[22] Greenland S (1989). Modeling and variable selection in epidemiologic analysis. Am J Public Health.

[23] Williams JW, Plassman BL, Burke J, Holsinger T, Benjamin S. Preventing Alzheimer’s Disease and Cognitive Decline. Agency for Healthcare Research and Quality (US); 2010. Hypertension.

[24] Iadecola C, Yaffe K, Biller J, Bratzke LC, Faraci FM, Gorelick PB (2016). Impact of hypertension on cognitive function: a scientific statement from the American Heart Association. Res Gate.

[25] Gavrila D, Antúnez C, Tormo MJ, Carles R, García Santos JM, Parrilla G (2009). Prevalence of dementia and cognitive impairment in southeastern Spain: the Ariadna study. Acta Neurol Scand.

[26] Beydoun MA, Beydoun HA, Gamaldo AA, Teel A, Zonderman AB, Wang Y (2014). Epidemiologic studies of modifiable factors associated with cognition and dementia: systematic review and meta-analysis. BMC Public Health.

[27] Khedr E, Fawi G, Abbas MAA, Mohammed TA, El-Fetoh NA, Al Attar G (2015). Prevalence of mild cognitive impairment and dementia among the elderly population of Qena governorate, upper Egypt: a community-based study. J Alzheimers Dis JAD.

[28] Valenzuela MJ, Sachdev P (2006). Brain reserve and dementia: a systematic review. Psychol Med.

[29] Valenzuela MJ, Sachdev P (2006). Brain reserve and cognitive decline: a non-parametric systematic review. Psychol Med.

[30] Valenzuela M, Brayne C, Sachdev P, Wilcock G, Matthews F (2011). Medical Research Council cognitive function and ageing study. Cognitive lifestyle and long-term risk of dementia and survival after diagnosis in a multicenter population-based cohort. Am J Epidemiol.

[31] Vuoksimaa E, Rinne JO, Lindgren N, Heikkilä K, Koskenvuo M, Kaprio J (2016). Middle age self-report risk score predicts cognitive functioning and dementia in 20–40 years. Alzheimers Dement Diagn Assess Dis Monit.

[32] Gorska-Ciebiada M, Saryusz-Wolska M, Ciebiada M, Loba J (2014). Mild cognitive impairment and depressive symptoms in elderly patients with diabetes: prevalence, risk factors, and comorbidity. J Diabetes Res.

[33] Lee SJ, Ritchie CS, Yaffe K, Stijacic Cenzer I, Barnes DE (2014). A clinical index to predict progression from mild cognitive impairment to dementia due to Alzheimer’s disease. PLoS One.

[34] Hebert LE, Scherr PA, McCann JJ, Beckett LA, Evans DA (2001). Is the risk of developing Alzheimer’s disease greater for women than for men?. Am J Epidemiol.

[35] Shigemori K, Ohgi S, Okuyama E, Shimura T, Schneider E (2010). The factorial structure of the mini-mental state examination (MMSE) in Japanese dementia patients. BMC Geriatr.

