# Supplementary material for: Cognitive impairment and hypertension in older adults living in extreme poverty: a cross-sectional study in Peru
Source: BMC Geriatr. 2017 Oct 26;17:250. doi: 10.1186/s12877-017-0628-8 (PMC5659043; doi:10.1186/s12877-017-0628-8)
Supplement: Additional file 1: — Table S1. Analysis using cognitive impairment as a count variable. Table S2. Treated vs untreated and controlled vs uncontrolled. (DOCX 21 kb) [file 12877_2017_628_MOESM1_ESM.docx]

**Additional file 1 Table S1 and S2**

| Table S1. Poisson regression results with cognitive impairment as a count variable | | | | | | | |  |
| --- | --- | --- | --- | --- | --- | --- | --- | --- |
| Characteristics | | Bivariate analysis | | |  | Multiple Regression | | |
|  |  | Mean ratio* | 95%CI | p |  | Mean ratio | 95%CI | p |
| Age (years) | |  |  |  |  |  |  |  |
|  | 65-70 | Ref. |  |  |  | Ref. |  |  |
|  | 71-80 | 0.94 | 0.93 - 0.95 | <0.001 |  | 0.95 | 0.94 - 0.96 | <0.001 |
| Sex |  |  |  |  |  |  |  |  |
|  | Female | Ref. |  |  |  | Ref. |  |  |
|  | Male | 1.07 | 1.06 - 1.09 | <0.001 |  | 1.06 | 1.04 - 1.07 | <0.001 |
| Educational level | |  |  |  |  |  |  |  |
|  | None | Ref. |  |  |  | Ref. |  |  |
|  | Primary or superior | 1.09 | 1.08 - 1.1 | <0.001 |  | 1.06 | 1.04 - 1.07 | <0.001 |
| Recent job | |  |  |  |  |  |  |  |
|  | No | Ref. |  |  |  | Ref. |  |  |
|  | Yes | 1.06 | 1.05 - 1.08 | <0.001 |  | 1.04 | 1.02 - 1.05 | <0.001 |
| Area |  |  |  |  |  |  |  |  |
|  | Urban | Ref. |  |  |  | Ref. |  |  |
|  | Rural | 0.97 | 0.96 - 0.99 | <0.001 |  | 0.97 | 0.96 - 0.98 | <0.001 |
| Diagnosed HT | |  |  |  |  |  |  |  |
|  | No | Ref. |  |  |  | Ref. |  |  |
|  | Yes | 1.01 | 0.99 - 1.02 | 0.392 |  | **1.02** | **1 - 1.03** | **0.020** |
| * Poisson regression models show ratio of means instead of mean differences | | | | | | |  |  |

| Table S2. Association between cognitive impairment and different hypertension outcomes | | | | | | | | |
| --- | --- | --- | --- | --- | --- | --- | --- | --- |
| Characteristics | | Bivariate analysis | | |  | Multiple Regression | | |
|  |  | PR | 95%CI | p |  | PR | 95%CI | p |
|  | Treatment |  |  |  |  |  |  |  |
|  | No | Ref. |  |  |  | Ref. |  |  |
|  | Yes | 0.93 | 0.31 - 2.79 | 0.898 |  | 0.80 | 0.25 - 2.54 | 0.702 |
|  | Controlled |  |  |  |  |  |  |  |
|  | No | Ref. |  |  |  | Ref. |  |  |
|  | Yes | 0.68 | 0.14 - 3.19 | 0.623 |  | 0.86 | 0.21 - 3.59 | 0.838 |
|  | Hypertension** |  |  |  |  |  |  |  |
|  | No | Ref. |  |  |  | Ref. |  |  |
|  | Yes | 0.85 | 0.51 - 1.40 | 0.52 |  | 0.76 | 0.47 - 1.24 | 0.275 |
| * Each hypertension outcome has been adjusted for age, gender, educational level, occupation, area | | | | | | | |  |
| ** Hypertension or blood pressure ≥140/90 | | | |  |  |  |  |  |
